# A novel nomogram for the preoperative prediction of sentinel lymph node metastasis in breast cancer

**DOI:** 10.1002/cam4.5503

**Published:** 2022-12-15

**Authors:** Xue‐fei Wang, Guo‐chao Zhang, Zhi‐chao Zuo, Qing‐li Zhu, Zhen‐zhen Liu, Sha‐fei Wu, Jia‐xin Li, Jian‐hua Du, Cun‐li Yan, Xiao‐ying Ma, Yue Shi, He Shi, Yi‐dong Zhou, Feng Mao, Yan Lin, Song‐jie Shen, Xiao‐hui Zhang, Qiang Sun

**Affiliations:** ^1^ Breast Surgery Department, Chinese Academy of Medical Sciences and Peking Union Medical College Peking Union Medical College and Hospital Beijing China; ^2^ Department of Thoracic Surgery National Cancer Center/National Clinical Research Center for Cancer/Cancer Hospital, Chinese Academy of Medical Sciences and Peking Union Medical College Beijing China; ^3^ Radiology Department, Xiangtan Central Hospital Hunan China; ^4^ Ultrasound Medicine Department Chinese Academy of Medical Sciences and Peking Union Medical College, Peking Union Medical College Hospital Beijing China; ^5^ Molecular Pathology Research Center, Department of Pathology Peking Union Medical College Hospital, Chinese Academy of Medical Sciences Beijing China; ^6^ Breast Surgery Department Baoji Maternal and Child Health Hospital Shaanxi China; ^7^ Breast Surgery Department Qinghai Provincial People's Hospital Qinghai China; ^8^ Breast Surgery Department Shanxi Traditional Chinese Medical Hospital Shanxi China

**Keywords:** axillary lymph node (ALN), breast cancer, nomogram, sentinel lymph node (SLN), ultrasound

## Abstract

**Background or Purpose:**

A practical noninvasive method to identify sentinel lymph node (SLN) status in breast cancer patients, who had a suspicious axillary lymph node (ALN) at ultrasound (US), but a negative clinical physical examination is needed. To predict SLN metastasis using a nomogram based on US and biopsy‐based pathological features, this retrospective study investigated associations between clinicopathological features and SLN status.

**Methods:**

Patients treated with SLN dissection at four centers were apportioned to training, internal, or external validation sets (*n* = 472, 175, and 81). Lymph node ultrasound and pathological characteristics were compared using chi‐squared and *t*‐tests. A nomogram predicting SLN metastasis was constructed using multivariate logistic regression models.

**Results:**

In the training set, statistically significant factors associated with SLN^+^ were as follows: histology type (*p* < 0.001); progesterone receptor (PR: *p* = 0.003); Her‐2 status (*p* = 0.049); and ALN‐US shape (*p* = 0.034), corticomedullary demarcation (CMD: *p* < 0.001), and blood flow (*p* = 0.001). With multivariate analysis, five independent variables (histological type, PR status, ALN‐US shape, CMD, and blood flow) were integrated into the nomogram (C‐statistic 0.714 [95% CI: 0.688–0.740]) and validated internally (0.816 [95% CI: 0.784–0.849]) and externally (0.942 [95% CI: 0.918–0.966]), with good predictive accuracy and clinical applicability.

**Conclusion:**

This nomogram could be a direct and reliable tool for individual preoperative evaluation of SLN status, and therefore aids decisions concerning ALN dissection and adjuvant treatment.

## Graphical abstract

This nomogram is using pathology and ultrasound parameters preoperatively to predict SLN metastasis. Cindex, calibration, discrimination, and clinical use of the nomogram show well in the training, internal and external validation sets.

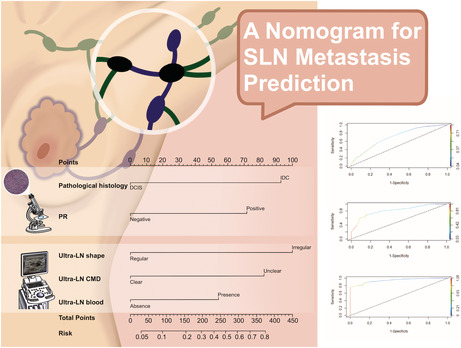

## 1 INTRODUCTION

Breast cancer is the most common malignant tumor afflicting women all over the world. In 2018, there were 2.28 million newly diagnosed cases.^1^, ^2^ Lymph node involvement and tumor size are the most important factors to determine the prognosis of breast cancer, and these remain crucial for strategizing individual treatments.^3^, ^4^, ^5^ Historically, axillary lymph node dissection (ALND) has been the reference standard for lymph node (LN) staging. However, for determining metastasis in axillary lymph nodes (ALNs), some recent studies found no significant differences in effectiveness between the sentinel lymph node (SLN) procedure and level I and II dissection.^6^, ^7^, ^8^, ^9^, ^10^, ^11^, ^12^, ^13^, ^14^, ^15^ What is more, because of fewer side effects (e.g., paresthesia, lymphedema, and restriction of movement), at many centers SLN dissection/biopsy has replaced ALND as the primary staging procedure.^16^, ^17^

For assessing the pathological status of ALNs in patients with stages I‐IIIA (T3N1M0) breast cancer, the National Comprehensive Cancer Network (NCCN)^18^ recommends SLN biopsy for surgical staging of clinically negative axilla. Yet, 20% to 60% of SLN^+^ patients have been found to lack non‐SLN involvement after ALND, indicating that these patients received unnecessary axillary treatment.^19^, ^20^, ^21^, ^22^ In a clinical setting with an SLN team of limited experience and a risk of false negatives, a lack of ultrasound (US) standard, and the possibility of unnecessary axillary treatment, there is increasing practical need to identify SLN status noninvasively. Selection criteria for preoperative SLN biopsy should be more precise than that offered by the NCCN.

Currently, differentiating benign from malignant nodes is aided by mammography, contrast‐enhanced US, positron emission tomography‐computed tomography, magnetic resonance imaging (MRI), and so on.^23^, ^24^, ^25^ Although some researchers consider that ALN‐US has low sensitivity, US‐guided biopsy of sonographically suspicious nodes somewhat increases the specificity up to 100%. The possibility of detecting some of these metastases and reducing the number of false negatives at sentinel node biopsy has renewed surgeons' interest.^3^, ^26^

Judging the SLN status of patients who have a suspicious ALN on US, but a negative clinical physical examination, is problematic. To differentiate patients with positive or negative SLNs based on ALN‐US features, this retrospective study compared ALN‐US data with pathological evidence, and a nomogram was constructed for predicting SLN metastasis.

## 2 METHODS

This study retrospectively analyzed patients who had a suspicious ALN at US, but a negative clinical physical examination, and were treated with SLN biopsy from 1 January 2014 to 31 December 2019 at the following medical centers: Peking Union Medical College and Hospital (PUMCH); Baoji Maternal and Child Health Hospital; Qinghai Provincial People's Hospital; and Shanxi Traditional Chinese Medical Hospital. Our retrospective study is based on real world data, according to clinical practice and NCCN guidelines.^18^ Patients were apportioned to following for analysis purposes: a training set of patients at PUMCH from 1 January 2014 to 1 January 2019 (*n* = 472); an internal validation set of patients at PUMCH from 1 January 2019 to 31 December 2019 (*n* = 175); and an external validation set (*n* = 81) of patients treated at 3 centers from 1 January 2019 to 31 December 2019 (described below).

Patients' demographics and clinical features were recorded, including age; ALN‐US findings; postoperative mass histological pathology, SLN status; type of breast surgery; and pathological risk factors. The latter consisted of lymphovascular invasion (LVI), nerve invasion, and infiltrative micropapillary carcinoma (IMPC). All the pathology was received at biopsy, that is, ductal carcinoma in situ (DCIS) included in our research was diagnosed on initial biopsy and found with invasive breast cancer at the time of the definitive surgical procedure. The SLN status, and SLN^+^ rate of the entire population, was analyzed. ALN‐US and pathological characteristics were compared between patients with positive and negative SLNs.

To select the training set, patients who had undergone SLN dissection (*n* = 2332) were initially reviewed. Potential subjects were excluded for the following reasons: combined negative clinical physical examination and negative US; received preoperative chemotherapy or endocrine therapy; with metastatic breast cancer or other special types of breast cancer; or were lost to follow‐up. Altogether, 1826 were excluded, and 472 patients at PUMCH were enrolled in the training set.

For the internal validation set, 175 patients with breast cancer at PUMCH were enrolled. In addition, 81 patients with breast cancer formed the external validation set, from Baoji Maternal and Child Health Hospital, Qinghai Provincial People's Hospital, and Shanxi Traditional Chinese Medical Hospital.

**FIGURE 1:**
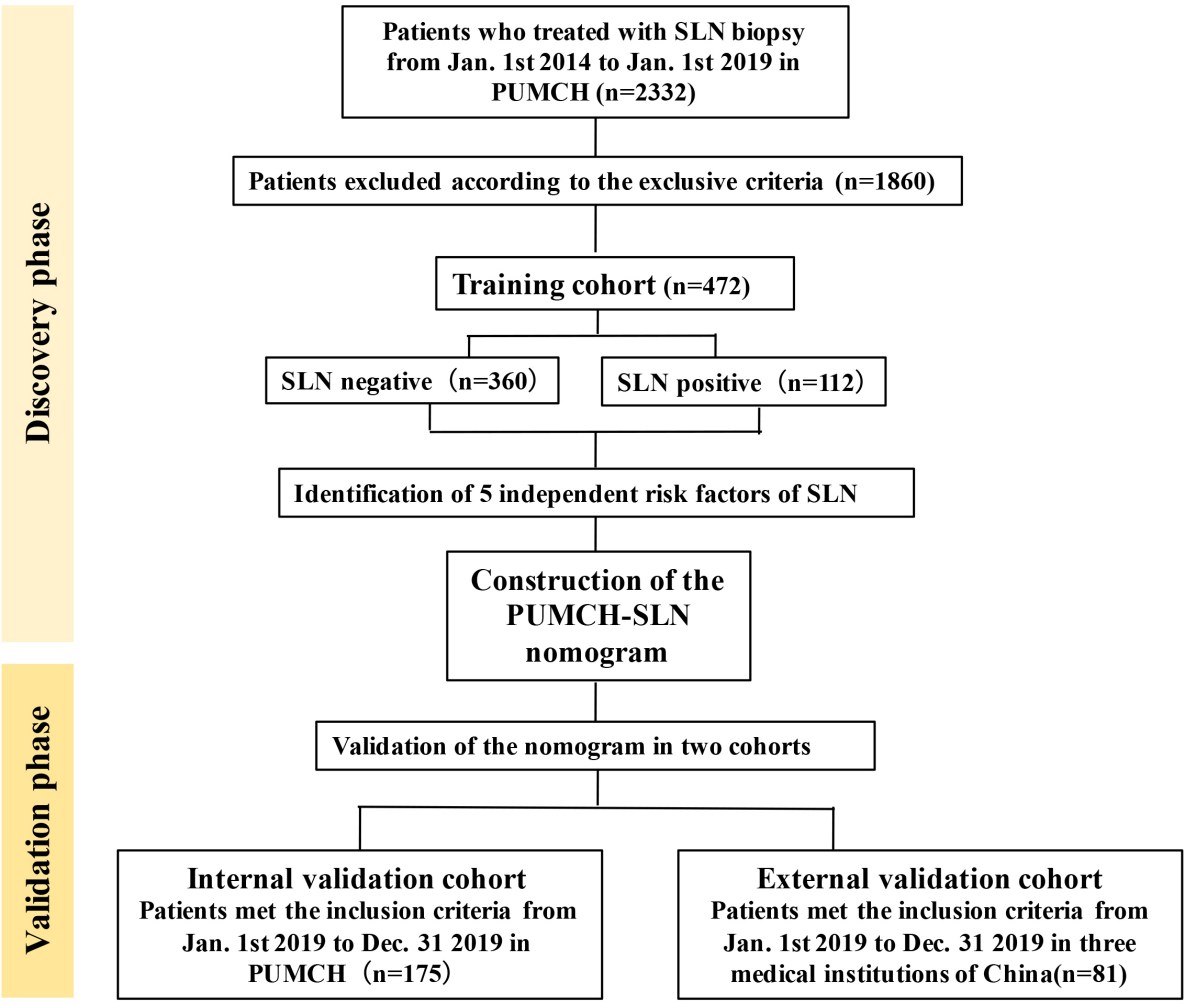
Flow chart of patient screening and exclusion criteria.

All the subjects were women. This study was performed according to the Declaration of Helsinki and approved by the Ethics Committee of the Peking Union Medical College and Hospital. The complete clinicopathological data of all patients were collected, and all the patients were followed up (Figure 1).

### 2.1 US techniques and characterization of LNs

ALN‐US scans were evaluated by 2 doctors (US specialists). The US specialists participating in this study are all experienced specialists who have received standardized training. The ultrasound examinations in all centers are performed by two ultrasound doctors at the same time. In case of disagreement, they will discuss and finally give a consensus. The US images were inverted from the ultrasonic system of each center. The size and morphological factors of Level I, II, and III LNs in the axillary and supraclavicular LNs on the side of breast tumors were recorded and evaluated. The morphological criteria included shape, corticomedullary demarcation (CMD), and blood flow on ALN‐US. Shape was described as regular or irregular. Asymmetric focal or diffuse cortical thickening >3 mm was classified as unclear ALN‐US CMD, and otherwise clear.^27^, ^28^

### 2.2 Surgical procedure, pathological evaluation, and adjuvant therapy

Lymphatic mapping of biopsied SLNs was performed using dual tracer, methylene blue, and indocyanine green (ICG). Surface localization was done by ICG, and then, we resected the observed blue‐stained LNs and enlarged LNs as the SLNs. ICG was used to reconfirm the resected SLNs. Every SLN was routinely analyzed via intraoperative frozen sections. Patients with negative SLNs were not given any further axillary treatment. ALN dissection was avoided in most of the patients who met the American College of Surgeons Oncology Group (ACOSOG) Z0011 criteria; otherwise, ALN dissection was performed.

### 2.3 Statistical analysis

#### 2.3.1 Clinical data

The clinicopathological and ALN‐US data of the patients were analyzed, and the patients were followed up. The last date of follow‐up was 1 January 2020. The main endpoint of follow‐up was death or distant metastasis. The mean follow‐up time was 42 months. The chi‐squared test (χ^2^) or Fisher's exact test was used to analyze an association between SLN metastasis and clinicopathological features. Comparisons of continuous variables (age, ALN‐US size, and Ki67) were performed using Student's *t*‐test. The Kaplan–Meier model was applied to analyze the survival rate of patients. The statistical analyses were two‐sided. *p* < 0.05 was considered a statistically significant difference. Statistical analyses were performed using SPSS software (version 24.0; IBM) and GraphPad Prism software (version 6.01).

#### 2.3.2 Construction of the nomogram

Associations between relevant clinicopathological variables plus ALN‐US with SLN status were estimated by multivariate logistic regression models; variables with *p* value <0.1 were included, and the odds ratios (ORs) and corresponding 95% confidence intervals (CIs) were calculated. The Hosmer–Lemeshow test was used for multivariate analyses, and a forest map to reflect the results of multivariate analysis. Based on the results of the multivariate analysis, independent variables were selected for inclusion in the nomograms to predict SLN metastasis, using statistical software (R 2.14.1, http://www.r‐project.org).^16^

#### 2.3.3 Discrimination and calibration of the nomogram

The nomogram was constructed using RMS software. The performance of the model was assessed through discrimination and calibration. Discrimination ability was calculated using concordance (C)‐statistics. The C‐statistic was applied to evaluate the concordance between the observed and predicted outcomes of the model, which is roughly equivalent to the receiver operating characteristic (ROC) curve. C‐statistics ranged from 0.5 and 1. To evaluate the calibration of the nomogram, a regression smoothing method was used to produce the calibration plots by bootstrapping with 1000 resamples, representing the association between the observed outcome frequencies and the predicted probabilities. The standard curve is a straight line passing through the origin of the coordinate axis with a slope of 1. The ROC curves were plotted using RROC software.

#### 2.3.4 Clinical use

Decision curve analysis was performed with remote direct memory access (RDMA) software and was used to evaluate the clinical value of the nomogram. The nomogram was constructed to estimate a net benefit for the prediction model by quantifying the net benefits at different threshold probabilities.^29^, ^30^

## 3 RESULTS

### 3.1 Clinical, US, and pathological characteristics

The training set comprised the data of 472 patients, with 360 and 112, respectively, SLN^−^ and SLN^+^. The patients were treated at PUMCH from 1 January 2014 to 1 January 2019. The internal validation cohort consisted of 175 patients, who were treated at PUMCH from 1 January to 31 December 2019. Eighty‐one patients made up the external validation cohort, and these patients were treated from 1 January to 31 December 2019 at Baoji Maternal and Child Health Hospital, Qinghai Provincial People's Hospital, and Shanxi Traditional Chinese Medical Hospital. The clinical, US, and pathological characteristics from the multicenter cohorts are listed in Table 1.

**TABLE 1:** Clinical characteristics of enrolled patients from the 3 multicenter cohorts^a^

			Training		Internal		Validation External	
				*p* value		*p* value		*p* value
Subjects, *n*			472		175		81	
Age, y			49.61 ± 11.59	0.087	50.82 ± 11.59	0.278	51.43 ± 9.56	**0.041**
ALN‐US	Size, cm		1.49 ± 0.62	0.359	1.53 ± 0.61	0.798	1.45 ± 0.63	0.072
	Shape	Regular	457 (96.8)	**0.034**	125 (71.4)	**<0.001**	48 (59.3)	**<0.001**
		Irregular	15 (3.2)		50 (28.6)		33 (40.7)	
	CMD	Clear	400 (84.7)	**<0.001**	140 (80.0)	**<0.001**	48 (59.3)	**<0.001**
		Unclear	72 (15.3)		35 (20.0)		33 (40.7)	
	Blood flow	Absent	244 (51.7)	**0.001**	67 (38.3)	0.009	42 (51.9)	**<0.001**
		Present	238 (48.3)		108 (61.7)		39 (48.1)	
Histological type^b^		DCIS	129 (27.3)	**<0.001**	21 (12.0)	**0.001**	6 (7.41)	**0.012**
		IDC	343 (72.7)		154 (88.0)		75 (92.59)	
Tumor size, cm		≤2	302 (64.0)	0.301	75 (42.9)	0.052	11 (13.6)	0.099
		2–5	159 (33.7)		99 (56.6)		62 (76.5)	
		>5	11 (2.3)		1 (0.6)		8 (9.9)	
ER		Positive	354 (75.0)	0.080	137 (78.3)	0.692	51 (63.0)	0.143
		Negative	118 (25.0)		38 (21.7)		30 (37.0)	
PR		Positive	322 (68.2)	**0.003**	120 (68.6)	0.457	46 (56.8)	0.034
		Negative	150 (31.8)		55 (31.4)		35 (43.2)	
Her‐2		Positive	122 (25.8)	0.049	39 (22.3)	0.356	5 (6.2)	0.624
		Negative	350 (74.2)		136 (77.7)		76 (93.8)	

They bold values mean *p* < 0.05.

Abbreviations: ALN‐US, axillary lymph node‐ultrasound; CMD, corticomedullary demarcation; DCIS, ductal carcinoma in situ; IDC, invasive ductal carcinoma; ER, estrogen receptor; PR, progesterone receptor.

^a^ Data are reported as mean ± standard deviation, or *n* (%).

^b^ At biopsy.

In the overall training cohort, 23.73% were SLN^+^; in the subgroup specifically with DCIS (at biopsy), this rate was 11.63%. The median age at diagnosis was 48 years (range, 16–85 year). The patients' ALN‐US and basic clinical pathological characteristics are listed in Table 2. The patients that were found SLN^+^ differed significantly from the SLN^−^ with regard to histology type (*p* < 0.001); ALN‐US shape, CMD, and blood flow (*p* = 0.034, <0.001, *p* = 0.001, respectively); progesterone receptor (PR) status (*p* = 0.003); and Her‐2 status (*p* = 0.049). There were no differences for LVI, nerve invasion, and IMPC (shown in Table 2). In the subgroup with invasive ductal carcinoma (IDC), the molecular subtypes of the 2 groups were statistically comparable (χ^2^ = 4.148, *p* = 0.246).

**TABLE 2:** ALN‐US and basic clinical pathological characteristics of the SLN^−^ and SLN^+^ patients in the training cohort^a^

			SLN (−)	SLN (+)	*p*
Subjects, *n*			360	112	
Age, y			50.12 ± 11.74	47.97 ± 10.98	0.087
ALN‐US	Size, cm		1.50 ± 0.62	1.44 ± 0.63	0.359
	Shape	Regular	352 (97.8)	105 (93.8)	0.034
		Irregular	8 (2.2)	7 (6.3)	
	CMD	Clear	319 (88.6)	81 (72.3)	<0.001
		Unclear	41 (11.4)	31 (27.7)	
	Blood flow	Absent	201 (55.8)	43 (38.4)	0.001
		Present	159 (44.2)	69 (61.6)	
Histological type^b^		DCIS	114 (33.6)	15 (16.1)	<0.001
		IDC	246 (66.4)	97 (83.9)	
Tumor size		T1	226 (62.8)	76 (67.9)	0.301
		T2	127 (35.3)	32 (28.6)	
		T3	7 (1.9)	4 (3.6)	
LVI		Presence	42 (11.7)	18 (15.1)	0.255
		Absence	318 (88.3)	94 (83.9)	
Nerve invasion		Presence	6 (1.7)	2 (1.8)	1.000
		Absence	354 (98.3)	110 (98.2)	
IMPC		Presence	7 (1.9)	6 (5.4)	0.090
		Absence	353 (98.1)	106 (94.6)	
ER		Positive	263 (73.1)	91 (81.3)	0.080
		Negative	97 (26.9)	21 (18.8)	
PR		Positive	233 (64.7)	89 (79.5)	0.003
		Negative	127 (35.3)	23 (20.5)	
Her‐2		Positive	101 (28.1)	21 (18.8)	0.049
		Negative	259 (71.9)	91 (81.3)	
Ki67, %			29.86 ± 23.49	29.96 ± 21.63	0.968

Abbreviations: ALN‐US, axillary lymph node‐ultrasound; SLN, sentinel lymph node; CMD, corticomedullary demarcation; DCIS, ductal carcinoma in situ; IDC, invasive ductal carcinoma; LVI, lymphovascular invasion; IMPC, infiltrative micropapillary carcinoma; ER, estrogen receptor; PR, progesterone receptor.

^a^ Data are reported as mean ± standard deviation, or *n* (%).

^b^ At biopsy.

### 3.2 SLN metastatic prediction model

#### 3.2.1 Independent variables of SLN ^+^ patients

**FIGURE 2:**
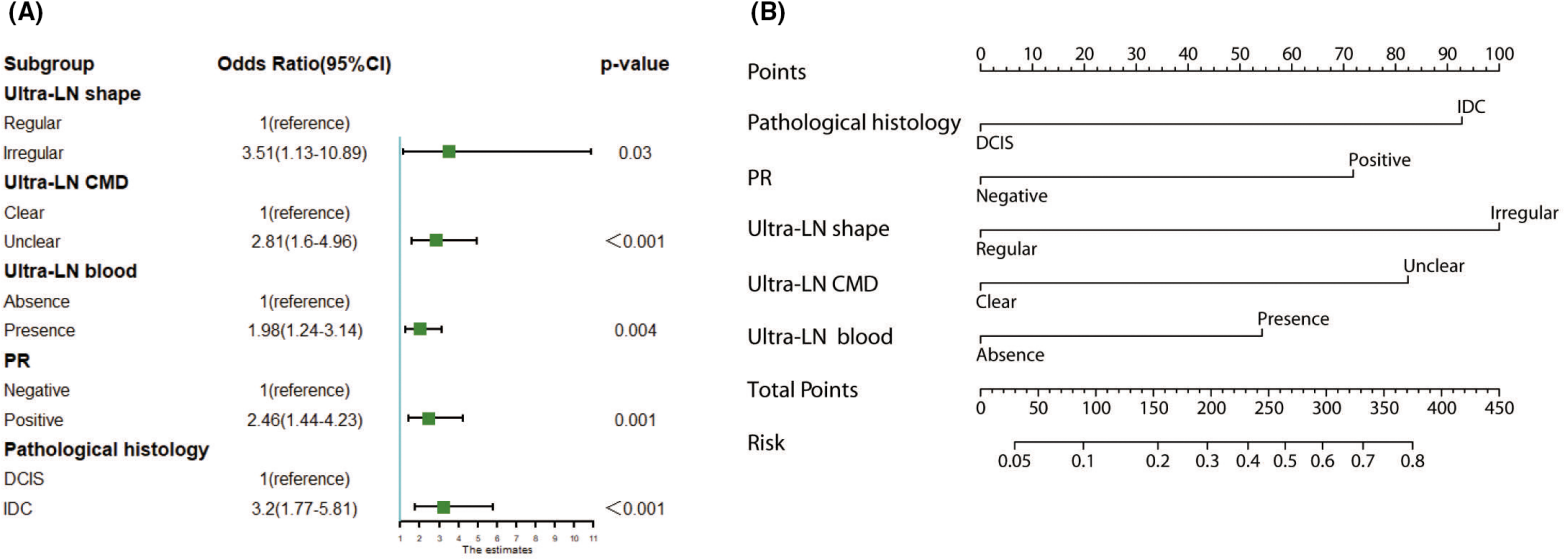
PUMCH‐SLN nomogram and risk factors. (A) Forest map of the multivariate analysis. (B) PUMCH‐SLN nomogram for the prediction of LN metastasis.

The univariate analysis indicated that histological type, PR, Her‐2, ALN‐US shape, ALN‐US CMD, and ALN‐US blood flow were significantly associated with SLN^+^ status. Based on the univariate analysis, variables with a difference of *p* < 0.1 were incorporated into the multivariate analysis (Figure 2A). With Hosmer–Lemeshow χ2 = 4.674, *p* = 0.700 > 0.05, this suggests that there is no statistical significance between the predictive value of the model and the actual observed value, and thus, the prediction model has a good calibration ability.

### 3.3 Predictive (PUMCH‐SLN) nomogram for breast cancer SLN metastasis

The multivariate logistic regression model found that histological type, PR status, ALN‐US shape, CMD, and blood flow were associated with an increased likelihood of SLN metastasis. Specifically: IDC histology (odds ratio [OR] 3.20; 95% CI 1.77–5.81; *p* < 0.001), PR positivity (OR 2.46; 95% CI 1.44–4.23; *p* = 0.001), irregular ALN‐US shape (OR 3.51; 95% CI 1.13–10.89; *p* = 0.030), unclear ALN‐US CMD (OR 2.81; 95% CI 1.60–4.96; *p* < 0.001), and blood flow on ALN‐US (OR 1.98; 95% CI 1.24–3.14; *p* = 0.004).

The logistic regression analysis was used to develop a nomogram, which we termed the PUMCH‐SLN nomogram. The PUMCH‐SLN nomogram combines pathology‐related factors (histological type, PR) and ALN‐US morphology (i.e., shape, CMD, and blood flow; Figure 2A). Each independent variable corresponds to a specific score when a linear line is draw straight upward to the score axis (Figure 2B). The total score refers to the sum of the score of each variate, which reflects the predicted probability of SLN metastasis by drawing a vertical line from the total score axis. Once the total is located, a vertical line is made between the total score and the final row. Row 8 predicts risk of SLN metastasis. For example, for the patient in Figure S1 with IDC breast cancer, PR‐positive, and ALN‐US with regular shape, unclear CMD, and blood flow (with scores of 100, 70, nil, 80, and 50, respectively), the total points equal a score of 300, which corresponds to a 60% risk of SLN metastasis.

### 3.4 Calibration and discrimination of the nomogram

**FIGURE 3:**
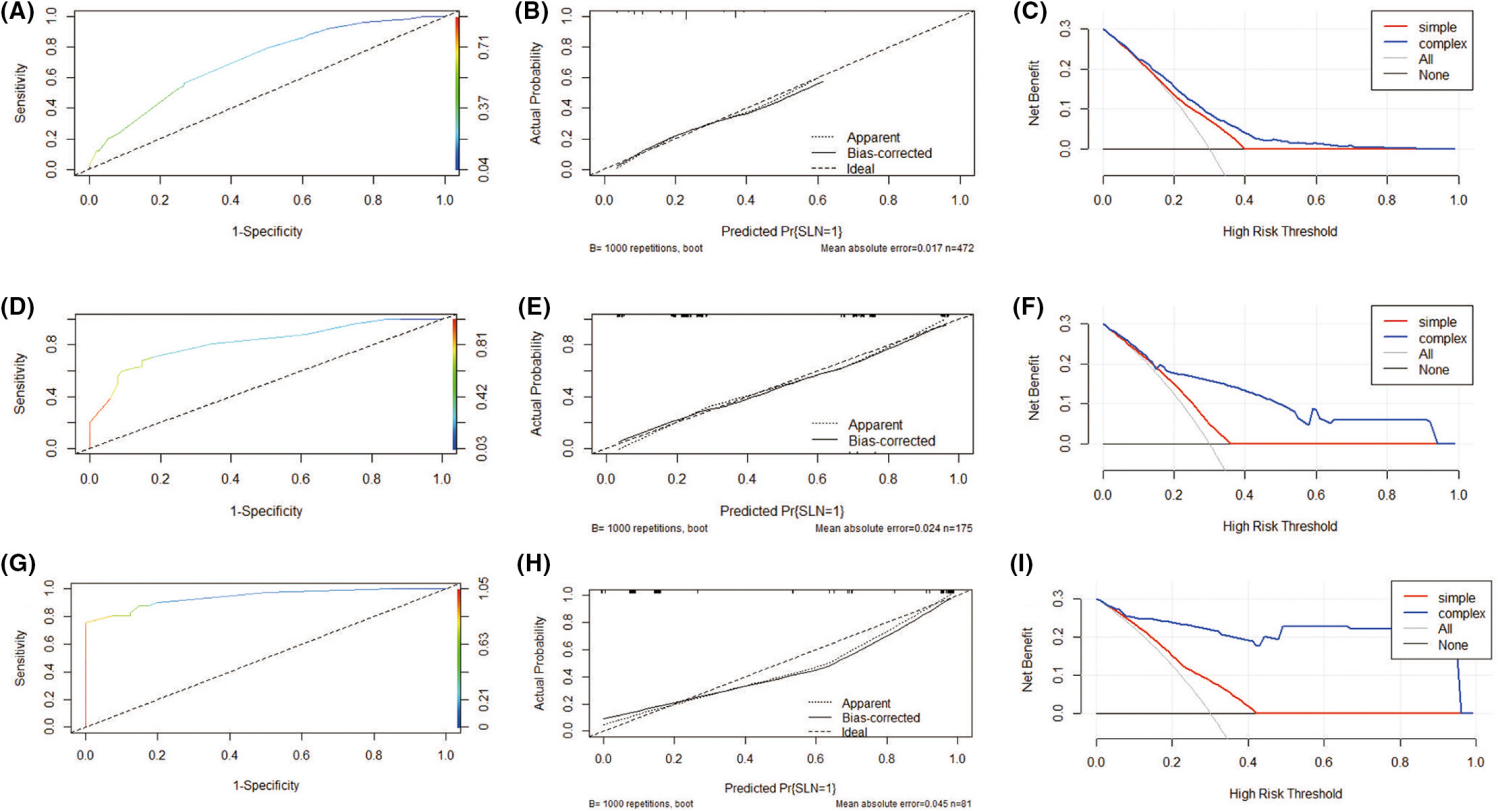
A + B + C: Calibration, discrimination, and clinical use of the nomogram in the training set. (A) Calibration curves of the PUMCH‐SLN nomogram. Calibration curves depict the calibration of the nomogram in terms of agreement between the predicted risk of SLN metastasis and actual SLN metastasis. The 45° oblique line represents a perfect prediction, and the dotted lines represent the predictive performance of the nomogram. The closer the dotted line fits to the ideal line, the better the predictive accuracy of the nomogram. After 1000 resampling, the predicted value of the model fits well with the actual observed value, which is near the 45° oblique line: mean absolute error 0.015; degree of discrimination C‐statistic 0.714 (95% CI: 0.688–0.740). (B) Plot showing the receiver operating characteristic curve of the PUMCH‐SLN nomogram. (C) Decision curve analysis for the PUMCH‐SLN nomogram. The *y*‐axis represents the net benefit. The blue line represents the PUMCH‐SLN nomogram. The red line represents the only use of pathological variables (histological types and PR status) to predict SLN metastasis. The black line represents the hypothesis that no patients had LN metastasis. The x‐axis represents the threshold probability. The decision curves showed that if the threshold probability is between 0.1 and 0.7, then using the PUMCH‐SLN nomogram to predict SLN metastasis adds more benefit than using only pathological factors. D + E + F: Calibration, discrimination, and clinical use of the nomogram in internal validation set. (D) Calibration curve of the PUMCH‐SLN nomogram internal validation group. C‐statistic 0.816 (95% CI: 0.784–0.849). (E) The receiver operating characteristic curve of the PUMCH‐SLN nomogram internal validation group. (F) The decision curve analysis of the PUMCH‐SLN nomogram internal validation group. G + H + I: Calibration, discrimination, and clinical use of the nomogram in external validation set. (G) Calibration curve of the PUMCH‐SLN nomogram external validation group. C‐statistic 0.942 (95% CI: 0.918–0.966). (H) The receiver operating characteristic curve of the PUMCH‐SLN nomogram external validation group. (I) Decision curve analysis of the PUMCH‐SLN nomogram external validation group.

The calibration plots showed acceptable consistency between the prediction via the nomogram and the actual observed outcome, with a mean absolute error = 0.015 (Figure 3A). The discrimination of the nomograms revealed good prognostic accuracy and clinical applicability, as indicated by the C‐statistic 0.714 (95% CI: 0.688–0.740; Figure 3B).

#### 3.4.1 Model validation

To verify further the accuracy of our model, 175 patients with suspected ALN metastasis based on US were selected from the patients with breast cancer as the internal validation group, treated from 1 January to 31 December 2019 at PUMCH. The external validation group comprised 81 patients treated from 1 January to 31 December 2019 at Baoji Maternal and Child Health Hospital, Qinghai Provincial People's Hospital, and Shanxi Traditional Chinese Medical Hospital. The C‐statistics of the internal and external validation groups were, respectively, 0.816 (95% CI: 0.784–0.849) and 0.942 (95% CI: 0.918–0.966). This further confirmed that the LN prediction model had good predictive accuracy and clinical applicability (Figure 3).

#### 3.4.2 Clinical use

The decision curve analysis for the PUMCH‐SLN nomogram is shown in Figure 3C,F,I. The simple model was constructed based on pathological type and PR status, and the complex model was constructed with all variables of the nomogram. According to previous research, the rate of LN metastasis is 25% to 28%.^9^ In the present study, within the threshold range of 0.1 to 0.7, the net benefit rate of the complex model is higher than that of the simple model. Thus, it was demonstrated that the PUMCH‐SLN nomogram is of great benefit to guide clinical decisions, and US can well supplement pathological factors for predicting SLN metastasis.

## 4 DISCUSSION

Herein, ALN‐US was evaluated relative to pathological characteristics for differentiating patients with positive or negative SLNs, and a PUMCH nomogram was constructed for predicting SLN metastasis. It was found that patients who were SLN^+^ were more likely to show the following: histological IDC at biopsy, PR‐positive status, ALN‐US with irregular shape, unclear CMD, and the presence of blood flow. The novel PUMCH nomogram is based on pathology and ALN‐US characteristics, and was demonstrated to have excellent ability to predict SLN metastasis, with an area under the C‐statistic curve (AUC) of 0.714. The PUMCH nomogram can be used to assist clinicians to predict SLN metastasis preoperatively, and thus may assist decisions regarding surgical strategy for patients with breast cancer.

This study provides preoperative axillary evaluation for all breast cancer patients, and not only for the specific types covered by the Z0011 or SOUND trial.^17^, ^31^ The nomogram is easy to generalize and is simple and intuitive to use. The AUC values were verified in validation groups, internally and externally, as 0.816 and 0.942, respectively. The calibration curve of the nomogram shows that the nomogram prediction is consistent with the actual metastasis rate. Such a nomogram is rare, based as it is on US and pathological parameters, supported by complete multicenter validation.

The current rise in SLN dissection has highlighted the need to predict LN metastasis and aid the selection and treatment of these patients. Scientists have reported many clinical characteristics that are related to LN metastasis, including multifocality, LN palpability, histological type, and LN status determined via radiomics.^9^, ^30^, ^32^, ^33^ In addition, Bevilacqua et al.^30^ found that patient age; tumor size, grade, and location; lymphovascular invasion; and estrogen receptor (ER) and PR status are related to LN metastasis.^34^ Other nomograms to assist LN prediction in breast cancer have been published.^35^

In recent years, more and more nomograms have been built using imaging data to preoperatively evaluate SLN metastasis, including breast MRI,^36^, ^37^ mammography,^38^ and chest CT.^39^, ^40^ Many have focused on a specific imaging examination combined with clinicopathological characteristics. Concerning CT, Yang et al.^39^ used deep learning signatures for preoperative prediction of SLN metastasis, with a validation AUC of 0.817. Huang et al.^41^ constructed a nomogram based on carcinoembryonic antigen (CEA) status, radiomics signature, and LN status depicted on CT. However, using CT for evaluating SLN status has not been widely promoted. Only one study has compared the three imaging methods (US, MRI, and mammography) for evaluating SLN metastasis,^38^ while research of each, respectively, has shown limited value. The above predictive models, which rely on CT and MRI, are not used widely by surgeons, and a nomogram based on US lacks detailed morphology. Most importantly, to our best knowledge a preoperative nomogram has not been constructed previously, specifically for patients with breast cancer who have a suspicious ALN on US, but simultaneously a negative clinical physical examination.

Others have predicted ALN in early breast cancer,^42^ or non‐SLN metastasis in patients during neoadjuvant chemotherapy.^43^ These models share similarities with ours, but are also fundamentally different. The nomogram of the present study has successfully resolved the questions raised above. Its greatest advantage is that LNs can be evaluated preoperatively using only the pathology based on biopsy and US morphological status. Its practicability and value are not only reflected in its convenience and rapid application, but also avoids false‐negative results from SLN biopsy to some degree. The false‐negative results from SLN biopsy range from 5.5% to 43%^44^ internationally. What is more, this nomogram can assist decisions to conduct further ALND in some complicated cases of breast cancer.

Although the Tenon, MSKCC, MDA, Mayo, Cambridge, and Stanford models^45^, ^46^ all assess the risk of non‐SLN metastasis using SLN and pathology, they are not designed especially for this very situation, and do not incorporate imaging features. The present PUMCH‐SLN nomogram provides a more detailed reference. The novel feature of our nomogram is that morphological status is incorporated with ALN‐US and is relevant for nearly all types of breast cancer treated in clinical practice.

To our best knowledge, there is no reliable evaluation and reporting standard for ALN, and the overall accuracy of ALN‐US remains controversial.^35^ Because the study of ALNs is strongly subjective, there is great variability in the judgments made by doctors specializing in US all over the world. In some studies,^27^, ^28^ LN size, or morphological findings, or both, were used as US criteria. However, recent studies^3^, ^26^ show that the morphological criteria of LNs on US are more important, especially the total replacement or eccentric hilus of the LN and hypoechoic cortex. Bedi et al.^19^, ^47^ emphasized that there is no difference between the size of benign and malignant LNs, and cortical morphological findings and hypoechoic cortex are more important than LN size. This is consistent with the present results. Whether the data are qualitative or quantitative, specific values across studies are not consistent. For example, cortical thickness >5 mm was identified as the cutoff for metastatic LNs, while diffuse cortical thickening >3 mm was reported as suspicious criteria with a specificity of 49% to 85%, and sensitivity of 96%, in predicting metastatic disease.^28^ For the present nomogram, diffuse cortical thickening >3 mm was considered suspicious, while other morphological indicators are subjective.

We wonder if ALN criteria in breast cancer might be incorporated into the Breast Imaging‐Reporting and Database System (BI‐RADS). Such criteria could include detailed evaluation of the eccentric placement, complete anechoic or hypoechoic appearance of the LN, axillary and echogenic hilus obliteration, asymmetric cortical thickening, and other possible quantitative evaluations gained from US ALN‐US. Our team is also developing another artificial intelligence‐assisted SLN prediction system. Based on a convolutional neural network, deep learning is gained via US images. In this way, metastatic LNs may be identified by ultrasonography with high sensitivity and positive predictive value, assisting in the stratification of patients with ALN metastasis, aiding in therapy planning and patient staging, and ultimately contributing to improvements in surgery strategy and survival rates.

In the research of Fidan et al.^28^ histopathological tumor size correlated with the primary tumor size. However, in the present study, the precise pathological T stage cannot be gained before surgery; only the tumor size may be determined by US. We were not able to differentiate SLN positivity by the tumor size shown on US (Table 1). Here, it should be noted that although some DCISs with microinvasion are large in diameter on US preoperatively, they have a lower risk of metastasis. Thus, though T was not intergreted in nomogram, a subgroup analysis of patients with IDC was conducted in the present study, using the chi‐squared and Fisher's exact tests. The results showed that, with χ^2^ = 6.333 and *p* = 0.032, there was a statistical difference. Hence, about 25% of women with seemingly pure DCIS on initial biopsy will be found with invasive breast cancer at the time of the definitive surgical procedure^48^ and thus will ultimately require ALN staging.^18^ Therefore, all patients with DCIS at biopsy in our study refer to DCIS patients with IDC finally, which is in accordance with the guidelines.

Besides, this study has other limitations. First, the sample size was limited, especially for external validation data from other institutions. Second, this is a retrospective study rather than prospective study. Thus, selection bias may have existed. Meanwhile, there is still room to improve the diagnostic efficiency of the model. What's more, ultrasound specialists' examination is subjective and inevitably biased. Therefore, we are conducting a prospective study to construct an artificial intelligence model.

## 5 CONCLUSIONS

In the present study, 1 in 4 patients with a suspicious ALN on US, but a negative clinical physical examination, had ALN metastasis. The PUMCH‐SLN nomogram constructed in this study shows that patients with IDC, PR‐positive status, and ALN‐US with irregular shape, unclear CMD, and presence of blood flow are more likely to be SLN^+^. The PUMCH‐SLN nomogram should be of great benefit to assist clinical decisions. US morphological characteristics are a highly credible reference to supplement pathology for predicting SLN metastasis. This nomogram could be a convenient practical preoperative tool to warn of false‐negative SLN. This nomogram could also assist in decisions about further treatment in some patients with complicated breast cancer.

## AUTHOR CONTRIBUTIONS

**Xuefei Wang:** Conceptualization (equal); writing – original draft (equal); writing – review and editing (equal). **Guo‐chao Zhang:** Methodology (equal). **Zhi‐chao Zuo:** Conceptualization (equal); formal analysis (equal). **Qing‐li Zhu:** Data curation (equal). **Zhen‐zhen Liu:** Data curation (equal). **Shafei Wu:** Data curation (equal). **Jia‐xin Li:** Supervision (equal). **Jian‐hua Du:** Supervision (equal). **Cun‐li Yan:** Conceptualization (equal); formal analysis (equal). **Xiao‐ying Ma:** Conceptualization (equal); formal analysis (equal). **Yue Shi:** Conceptualization (equal); formal analysis (equal). **He Shi:** Conceptualization (equal); formal analysis (equal). **Yidong Zhou:** Project administration (equal). **Feng Mao:** Project administration (equal). **Yan Lin:** Project administration (equal). **Song‐jie Shen:** Project administration (equal). **Xiaohui Zhang:** Project administration (equal). **Qiang Sun:** Project administration (equal).

## FUNDING INFORMATION

This work was supported by Fundamental Research Funds for the Central Universities (No. 3332021012) and CAMS Innovation Fund for Medical Sciences (CIFMS) (No. 2021‐I2M‐C&T‐B‐018), National High Level Hospital Clinical Research Funding(2022‐PUMCH‐A‐018), and Tsinghua University‐Peking Union Medical College Hospital Initiative Scientific Research Program (No. 2019Z).

## CONFLICT OF INTEREST

Each author declared that he/she has no conflict of interest.

## ETHICS APPROVAL AND CONSENT TO PARTICIPATE

This study was approved by the ethics committee of Peking Union Medical College and Hospital. All procedures performed in studies involving human participants were in accordance with the ethical standards of the institutional and/or national research committee and with the 1964 Helsinki declaration and its later amendments or comparable ethical standards.

## CONSENT FOR PUBLICATION

All data published here are under the consent for publication. Written informed consent was obtained from all individual participants included in the study.

## Supporting information


Figure S1

## Data Availability

The datasets generated and analyzed during the present study are available from the corresponding author on reasonable request.
