# Supplementary figures and images for: A novel nomogram for the preoperative prediction of sentinel lymph node metastasis in breast cancer
Source: Cancer Med. 2022 Dec 15;12(6):7039–50. doi: 10.1002/cam4.5503 (PMC10067027; doi:10.1002/cam4.5503)

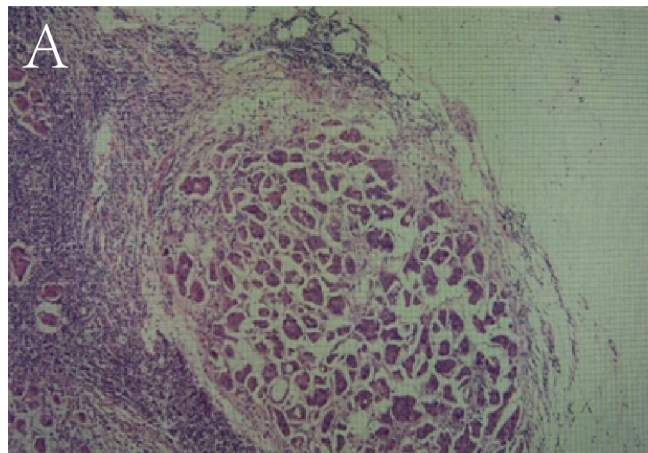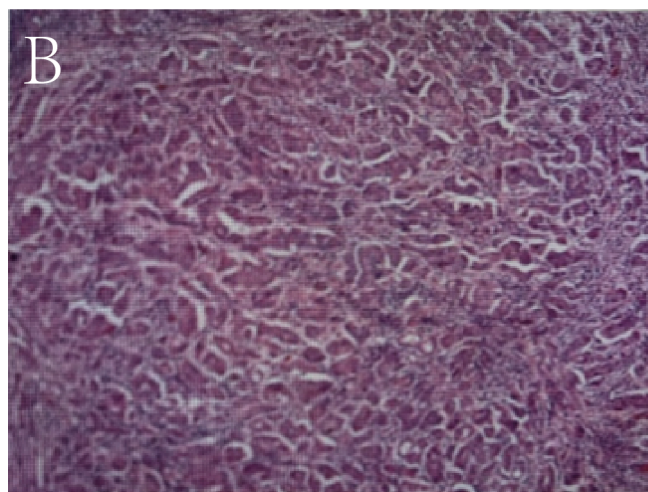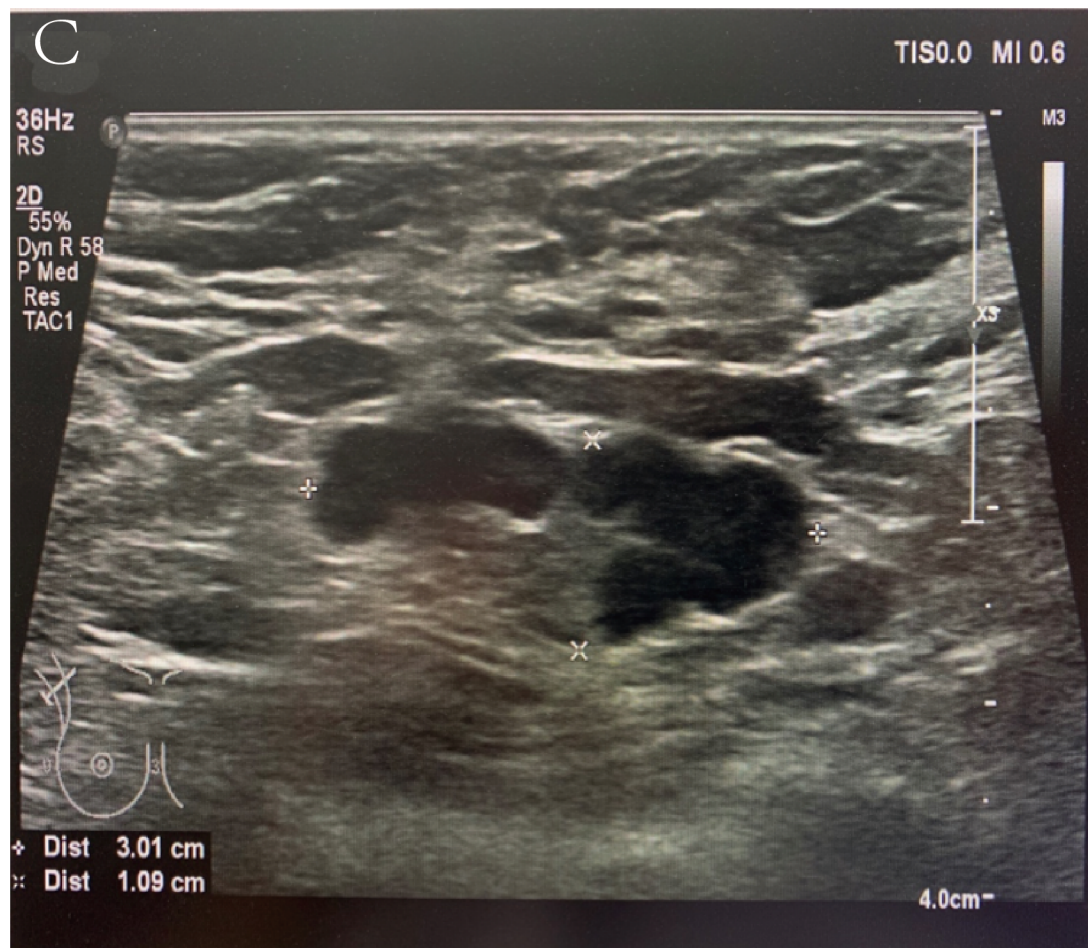

Supplement: Supplementary file 1 — Figure S1 [file CAM4-12-7039-s001.pdf]
